# Anatomy of the Female Organs

**Published:** 1859-09

**Authors:** M. Ronget


*Anatomy of the Female Organs.* By M. RONGET.

M. Charles Ronget's interesting researches on the anatomy of the female organs are summed up in the following propositions;

1 In woman the body of the uterus presents the structure of an erectile organ, of a true corpus spongiosum.

2. That to the ovary also an erectile bulb is attached.

3. That in all the classes of vertebrata, and especially in all mammifera, a special muscular apparatus embraces the oviduct and ovary, and effects their adaption to each other.

4. That the fasiculi of the ovario-tubal muscular membranes (mesoarium and mesometrium) have such relations with the corpora spongiosa, and especially with their efferent sinuses, that, at the moment of contraction, the meshes of the network in the midst of which run the venous channels, contracting in every direction, these must necessarily be compressed, and the discharge of blood more or less completely stopped.

5. That the contraction of the ovario-tubal muscular apparatus persisting during the whole period of ovulation, the obstacle to the passage of blood and the erection of the corpora spongiosa of the uterus and ovary, which is the result, have the same duration.

6. That menstruation coinciding also, on the other hand, with ovulation, it is natural to consider it as the immediate consequence of the erection of the uterus; a true menstrual hemorrhage, moreover, never presenting itself but where the uterus possesses a truly erectile structure.

7. That if sexual excitation, as seems probable, can determine the erection of the uterus and ovary, it is easy to account by that fact for the coincidence of the periods of menstruation and ovulation.

